# Establishment of sperm cryopreservation and *in vitro* fertilisation protocols for rats

**DOI:** 10.1038/s41598-019-57090-7

**Published:** 2020-01-09

**Authors:** Naomi Nakagata, Nobuyuki Mikoda, Satohiro Nakao, Ena Nakatsukasa, Toru Takeo

**Affiliations:** 1 Center for Animal Resources and Development, Kumamoto University, 2-2-1 Honjo, Chuo-ku, Kumamoto 860-0811 Japan; 2 Department of Animal Model Development, Brain Research Institute, Niigata University, 1-757 Asahimachidori, Chuo-ku, Niigata 951-8585 Japan

**Keywords:** Genetic models, Genetic engineering

## Abstract

Recently, genome-editing tools have come into common use in the field of rat research, and consequently, many genetically modified rat strains have been preserved and archived as frozen embryos. Although there have been many reports published on the topic of rat sperm cryopreservation, no report has yet provided satisfactory and acceptable protocols for the cryopreservation of rat sperm. In this study, we developed methods for both the cryopreservation of transgenic rat sperm and *in vitro* fertilisation using frozen sperm, which yielded high fertilisation rates.

## Introduction

Over the past ten years, genome-editing techniques have been used to produce many genetically modified rat strains^1^. Consequently, the cryobanking of gametes and embryos from these strains is rapidly becoming an important issue. Frozen embryos and sperm from many rat strains have been preserved in rat resource centres worldwide, including the National BioResource Project - Rat, Kyoto University, Japan; the Rat Resource & Research Center, University of Missouri, USA; and the Gene Editing Rat Resource Center, Medical College of Wisconsin, USA.

In general, embryo freezing is a reliable method for preserving rat strains. Using the conventional embryo freezing method, 400–500 embryos per strain are required from the oviducts of 30–50 mated females. Moreover, if only a few genetically modified males are available to be used for mating, it would take from 2 to 4 months to obtain sufficient embryos for cryopreservation because the number of females used for mating at any one time is limited and the males have to mate with females multiple times. On the other hand, a large number of spermatozoa can be cryopreserved immediately after being collected from the epididymides of males. Thus, sperm freezing is a much simpler, more efficient and more economical alternative to embryo freezing for preserving genetically modified rat strains.

Since the publication in 2001 of the first successful report concerning the cryopreservation of rat sperm^2^, many papers on the topic have been published^3–13^. However, only one of those papers has reported the successful production of pups derived from embryos that were obtained via *in vitro* fertilisation using frozen rat sperm^5^. Meanwhile, satisfactory and acceptable protocols for rat sperm cryopreservation and subsequent IVF have not yet been established.

Recently, we established a rat sperm cryopreservation method that results in high fertilisation rates via *in vitro* fertilisation using frozen/thawed sperm. In this paper, we restrict ourselves to a description of the detailed procedure routinely used for freezing rat sperm.

## Results

### *In vitro* fertilisation of either fresh or frozen rat sperm

**Figure 1:**
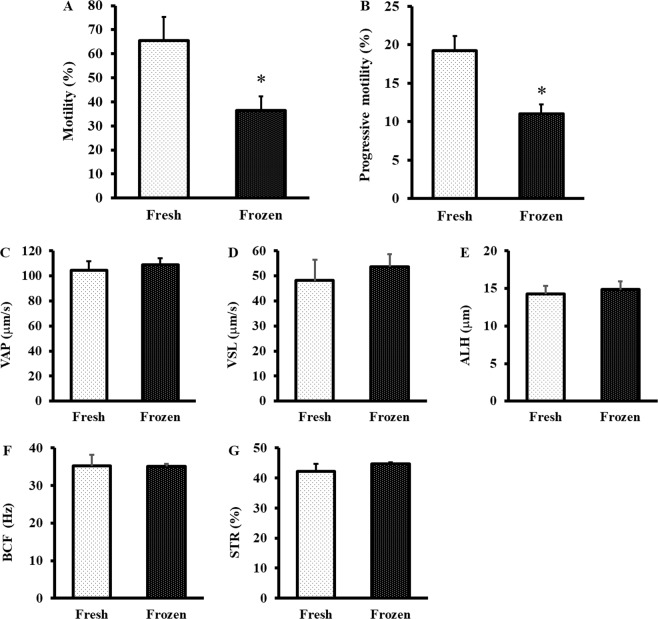
Motile parameters of fresh and cryopreserved rat sperm Parameters of sperm motility were analysed by a computer-assisted sperm analyser. (**A**) Motility is the ratio of sperm moving 5 μm/s to total sperm. (**B**) Progressive motility is the ratio of sperm moving at 50 μm/s and with straightness greater than 50% to total sperm. (**C**) Path velocity (VAP) is the average velocity of the smoothed sperm path. (**D**) Progressive velocity (VSL) is the average velocity measured in a straight line from the beginning to the end of the sperm track. (**E**) Lateral amplitude (ALH) is the mean width of the head oscillation as the sperm swims. (**F**) Beat frequency (BCF) is the frequency of sperm heads crossing the average sperm path in either direction. (**G**) Straightness (STR) is a measure of the departure of the average sperm path from a straight line (ratio of VSL/VAP). Values are given as the mean ± SD (n = 4). *p < 0.05 compared with fresh sperm.

High fertilisation rates were achieved with both fresh and frozen rat sperm (Table 1). However, the fertilisation rates achieved using cryopreserved sperm were lower than the rates achieved with fresh sperm (P < 0.05). Fresh sperm also had higher percentages of motile and progressive motile sperm than frozen sperm (Fig. 1).

**Table 1:** Fertilisation rate of oocytes inseminated using fresh or frozen rat sperm.

Sperm	No.	No. ofinseminated oocytes	No. offertilised oocytes	Fertilisation rate (%)
Fresh	1	198	185	93.4
	2	183	170	92.9
	3	195	181	92.8
	4	182	174	95.6
	Total	758	710	93.7 ± 1.3
Frozen	1	161	135	83.9
	2	191	160	83.8
	3	201	161	80.1
	4	196	159	81.1
	Total	749	615	82.1 ± 1.9*

*P < 0.05 compared with fresh sperm.

### Developmental ability of fertilised rat oocytes *in vitro* and *in vivo*

**Figure 2:**
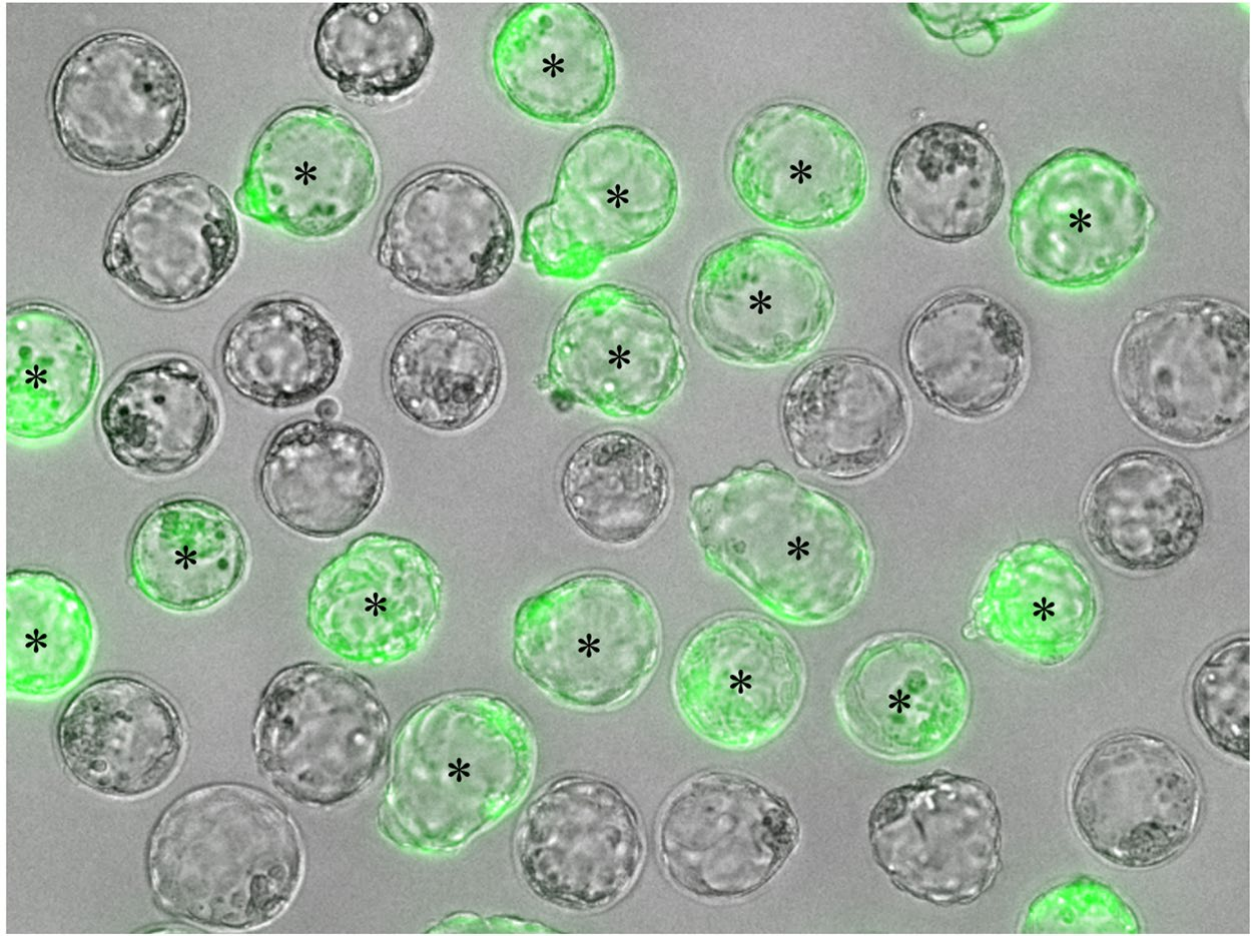
Blastocysts derived from *in vitro* fertilisation using cryopreserved rat sperm. Wild-type blastocysts and GFP-positive blastocysts (green) are shown. GFP-positive blastocysts are labelled with asterisks.

**Figure 3:**
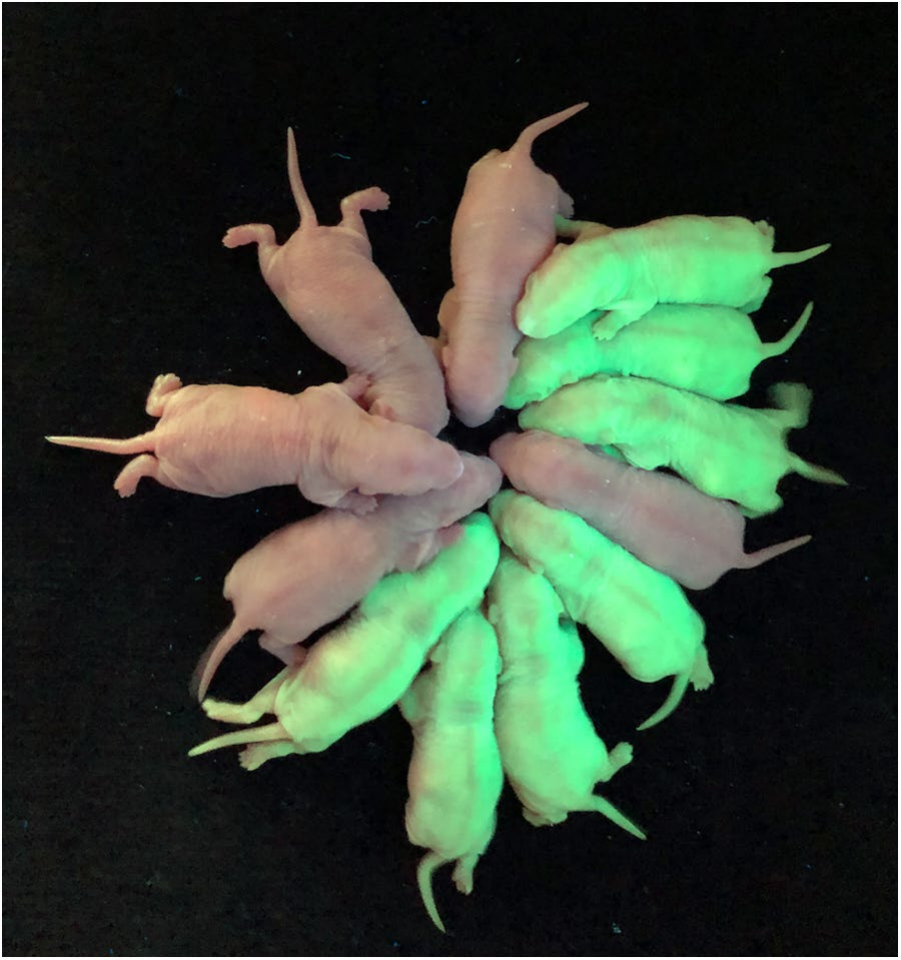
Live pups derived from *in vitro* fertilisation with cryopreserved rat sperm. Wild-type pups and GFP-positive pups (green) are shown.

Almost all fertilised oocytes developed to 2-cell embryos 28 hours after insemination. More than 60% of 2-cell embryos derived from cryopreserved and fresh sperm developed to blastocysts, half of which were blastocysts with green fluorescent protein (GFP) signals (Table 2 and Fig. 2). A total of 46 normal pups (26 pups with GFP signals) and 48 normal pups (24 pups with GFP signals) were obtained from cryopreserved and fresh sperm, respectively (Table 3 and Fig. 3). There were no differences between the development of the fertilised oocytes derived from either cryopreserved or fresh sperm either *in vitro* or *in vivo*.

**Table 2:** *In vitro* developmental ability of 2-cell embryos derived from fresh or cryopreserved rat sperm.

Sperm	No.	No. of 2-cell embryos	No. of blastocysts	Developmental rate (%)	No. of blastocysts with GFP	Success rate (%)
Fresh	1	100	58	58	27	46.6
	2	100	63	63	30	47.6
	3	100	65	65	33	50.8
	4	100	71	71	35	49.3
	Total	400	257	64.3 ± 5.4	125	48.6 ± 1.9
Frozen	1	100	72	72	36	50.0
	2	100	57	57	28	49.1
	3	100	64	64	29	45.3
	4	100	53	53	34	64.2
	Total	400	246	61.5 ± 5.4	127	51.6 ± 8.3

Developmental rate = no. of blastocysts/no. of 2-cell embryos × 100.

Success rate = no. of blastocysts with GFP/no. of blastocysts × 100.

**Table 3:** *In vivo* developmental ability of fertilised oocytes derived from fresh or frozen rat sperm.

Sperm	No.	fertilised oocytes transferred	No. of pups	Birth rate (%)	No. of pups with GFP	Success rate (%)
Fresh	1	20	13	65.0	6	46.2
	2	20	12	60.0	5	41.7
	3	20	13	65.0	6	46.2
	4	20	10	50.0	7	70.0
	Total	80	48	60 ± 7.1	24	50.0 ± 12.8
Frozen	1	20	12	60.0	8	66.7
	2	20	12	60.0	7	58.3
	3	20	10	50.0	2	20.0
	4	20	12	60.0	9	75.0
	Total	80	46	57.5 ± 5.0	26	56.5 ± 24.3

Birth rate = no. of pups/no. of fertilised oocytes transferred × 100.

Success rate = no. of pups with GFP/no. of pups × 100.

## Discussion

The current study is the first report to show that transgenic rat spermatozoa can be successfully cryopreserved and that the oocytes fertilised by cryopreserved spermatozoa can develop into normal offspring after *in vitro* fertilisation and transfer of the fertilised oocyte.

Although the success of *in vitro* fertilisation using fresh sperm was demonstrated by Toyoda *et al*. in 1974^14^, there were no successful cases of *in vitro* fertilisation using frozen sperm for many years after that. It was 2009 before the first account of successful *in vitro* fertilisation using frozen Wistar rat sperm, which was reported by Seita *et al*.^5^. Their data showed that when isobutylmethylxanthine (IBMX)-treated frozen/thawed sperm were used for *in vitro* fertilisation, the rates of pronuclear formation and blastocyst formation were significantly higher (pronuclear formation: 50%, blastocyst formation: 20%, birth rate: 49%) than what they achieved when frozen/thawed sperm were used without IBMX treatment. However, since then, there have been no papers showing that frozen sperm can retain sufficient sperm motility to produce pups via *in vitro* fertilisation and embryo transfer.

In the current study, we addressed the problem of poor reproducibility of rat sperm cryopreservation and *in vitro* fertilisation. We have combined the best of our knowledge and experience in rat reproductive technology. We demonstrated fertilisation rates of more than 80% using cryopreserved transgenic rat sperm (Table 1), and 60% of 2-cell embryos developed to the blastocyst stage (Table 2). Furthermore, 58% of transferred fertilised oocytes developed into pups (Table 3).

**Figure 4:**
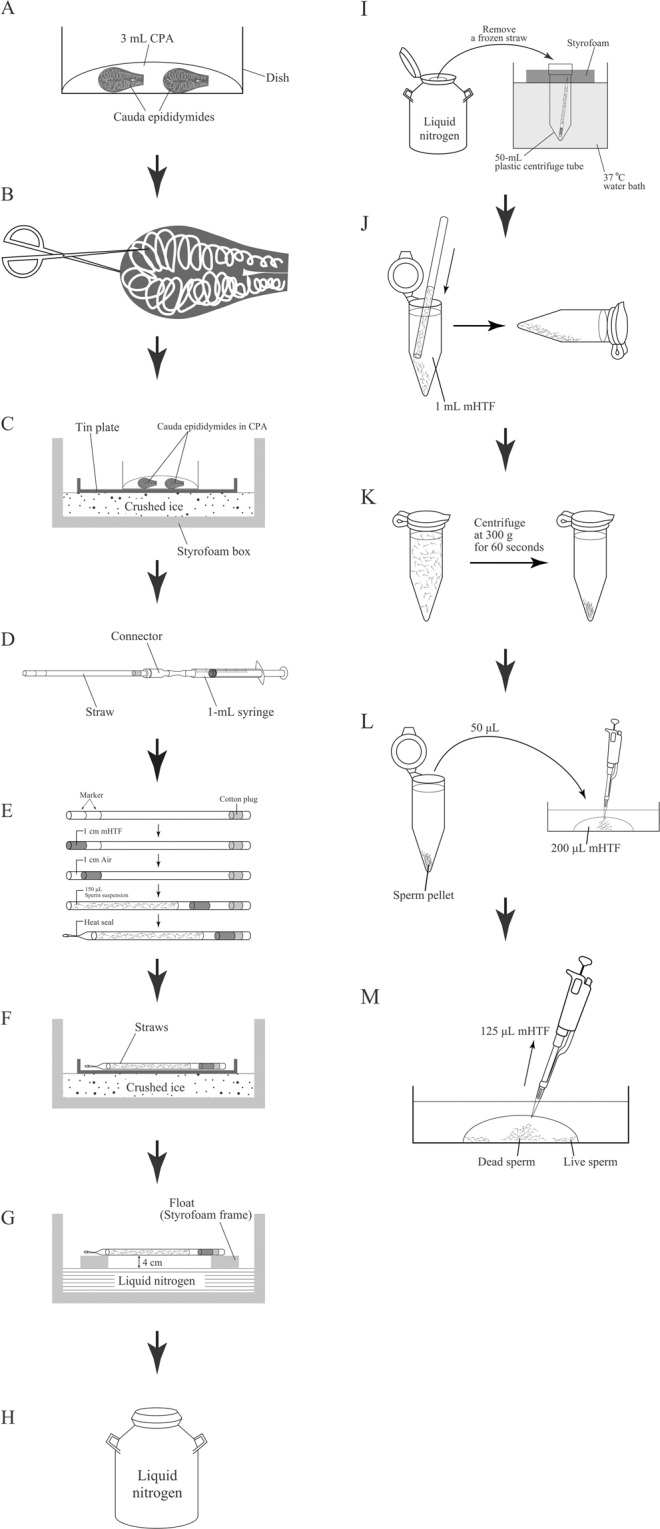
Sperm cryopreservation and *in vitro* fertilisation in rat.

Rat sperm is known to be extremely sensitive to environmental changes, such as shifts in viscosity and osmotic stress^15,16^. As such, if a cryopreservation agent (CPA) containing sperm is diluted immediately after thawing, the cryopreserved sperm may be damaged. In our previous study, we found that the fertilising ability of mouse sperm that were diluted using mHTF immediately after thawing was much lower than the rate of sperm that were diluted slowly^17^. The rat sperm CPA in the current study has high viscosity and an osmolarity of 370–380 mOsm. On the other hand, mHTF used for dilution has low viscosity and an osmolarity of 300–310 mOsm. Thus, we think that it is very important to preincubate the straw containing the sperm suspension in a 37 °C water bath for fifteen minutes (Fig. 4I) and to slowly dilute the cells using the swim-up method (Fig. 4J) after thawing; both strategies are described in this paper because they prevent sharp changes in viscosity and osmolarity between the CPA and the diluent. In addition, suppressing the movement of sperm at 0 °C before freezing may be another important factor (Fig. 4C–F). Further investigation of the above factors is required to comprehend the improvement of quality of cryopreserved rat sperm described here.

Over the past ten years, many transgenic and targeted mutant rats have been produced worldwide^1^. Moreover, even more genetically modified rats will be produced using genome-editing techniques in the near future^18^. However, rats are approximately ten times larger than mice, and the number of rats that can be kept in an animal room is limited. Thus, we would need a large capital investment and substantial space to keep hundreds of rats at our centre, and preservation of transgenic rat strains will become a major issue in the near future. Moreover, opportunities to exchange these rat strains will increase rapidly because of collaborations between institutes. As we have mentioned previously, a large number of spermatozoa can be collected from just one male. Ergo, if the sperm collected from 2–3 males is cryopreserved, we can produce more than 300 pups at a later date using that sperm via *in vitro* fertilisation and embryo transfer. This method may facilitate a reduction in the number of rats required to maintain rat strains. Therefore, we strongly believe that sperm freezing represents an extremely powerful tool for storing a large number of rat strains with induced mutations, and we further believe that we will see application of the technique in the exchange of mutant strains between labs worldwide.

## Materials and Methods

### Animals

Male transgenic rats (SD-Tg(CAG-EGFP)4Osb) were purchased from Slc Japan (Shizuoka, Japan) (http://www.anim.med.kyoto-u.ac.jp/NBR/strains/Strains_d.aspx?StrainID=559&s_geneAffected=GFP&s_References=GFP&s_livingAnimals=1) and were used as sperm donors at 11–12 weeks of age; Slc:SD female rats (purchased from Slc) were used as oocyte donors at 4–5 weeks of age. Meanwhile, Crl:CD (SD) female rats purchased from Charles River Japan (Kanagawa, Japan) were used as recipients of fertilised oocytes at 10–15 weeks of age. All animals were housed under a twelve-hour dark/light cycle (light from 07:00 to 19:00) at 22 °C ± 1 °C, with ad libitum access to food and water. The Animal Care and Use Committee of Kumamoto University School of Medicine approved the protocols for animal experiments and all methods were performed in accordance with the relevant guidelines and regulations.

### Sperm cryoprotective agent

Preparation of a cryopreservation agent (CPA) for sperm was carried out essentially following the method described by Nakatsukasa *et al*.^2,3^. A solution of 8% (w/v) lactose (Sigma, St Louis, MO) and 23% (v/v) chicken egg yolk, with 1,000 units/mL of penicillin G and 1 mg/mL of streptomycin sulfate, was mixed in distilled water. After adjusting the pH of the solution to 7.4 with 10% tris aminomethane solution, the solution was centrifuged at 1,600 g for 15 minutes, and the upper layer of the solution was collected. Then, 0.7% Equex Stm (ES; Nova Chemical Sales, Inc., Scituate, Mass.) and 0.1% ATP (Adenosine 5′-triphosphate disodium salt hydrate Grade II; Sigma) were added to the solution^7,8^, and the solutions was mixed on a stirring plate. Finally, the CPA was divided into aliquots and stored in a freezer at −20 °C before use.

### Sperm freezing

In each experiment, one male transgenic rat was used for sperm freezing.

Figure 4 summarises the sperm freezing process.

1. A male rat was euthanised via cervical dislocation, and then its two cauda epididymides were removed aseptically and transferred into a 300 μL drop of CPA in a 35 mm culture dish at room temperature (Fig. 4A).
2. Ten to twelve deep cuts were made in the epididymides using scissors under a microscope (Fig. 4B).
3. The dish was placed on a tin plate, which was set on top of crushed ice, and the tin was incubated there for ten minutes. (Fig. 4C).
4. During equilibration of the sperm in the CPA, a connector with a 1 mL syringe was attached to the end of a straw (Fig. 4D) and placed on the aforementioned tin plate. Fifteen straws can be prepared from the two epididymides of a single male.
5. A total of 30 μL mHTF (KYUDO Co. Ltd, Saga, Japan) at 0 °C was aspirated carefully into the straw along with 10 mm air. Then, 150 μL sperm suspension was aspirated, and the plunger of the syringe was drawn up until the 30 μL mHTF reached the cotton plug in the straw. The tip of the straw was then sealed using an impulse sealer (Fig. 4E).
6. Fifteen sealed straws were placed on a tin plate, which was set on top of crushed ice and incubated for thirty minutes (Fig. 4F).
7. The straws were transferred to a float (Styrofoam frame). The Styrofoam frame holding the straws was floated promptly on liquid nitrogen inside a Styrofoam box and was kept there for ten minutes (Fig. 4G).
8. After ten minutes, the straws were immersed in liquid nitrogen. The straws were then transferred to a triangular cassette, which was stored in the liquid nitrogen tank for 4–8 weeks (Fig. 4H).

### Sperm thawing

In each experiment, 3–4 of the fifteen cryopreserved straws were used for sperm thawing.

1. One millilitre of mHTF was placed in a 1.5 mL tube, and the medium was equilibrated in an incubator (37 °C, 5% CO_2_) for 30 minutes before use.
2. A frozen straw was removed from the liquid nitrogen tank and placed in the floating container, after which it was preincubated in a 37 °C water bath for 15 minutes to thaw the cell (Fig. 4I).
3. The straw was removed from the water bath and dried using a paper towel.
4. The straw was cut in the area between the sperm suspension and the seal and then placed in a tube containing mHTF. Then, after cutting off the cotton plug at its end, the straw was inserted into the straw connector, and only the sperm suspension was transferred into the bottom of the tube by pushing down the plunger of the 1 mL syringe. The tube was then laid on its side and kept in a humidified incubator (37 °C, 5% CO_2_) for 30 minutes (Fig. 4J).
5. After 30 minutes, the tube was inverted slowly 2–3 times and then centrifuged at 300 g for 60 seconds (Fig. 4K). Then, 50 μL sediment containing sperm pellets was sucked from the bottom of the tube using a 200 μL pipette with a large opening (Cat. No. 4290–00 S, Funakoshi Co., Ltd. Tokyo, Japan). The sediment was transferred to a 200 μL drop of mHTF covered with paraffin liquid in a culture dish (Fig. 4L).
6. After 30 minutes, 125 μL medium containing dead sperm was removed from the mHTF drop (Fig. 4M), and the sperm suspension was preincubated for two hours prior to insemination.

### ***In vitro*** fertilisation and development of fertilised oocytes

In each experiment, *in vitro* fertilisation was carried out using fresh sperm and cryopreserved sperm taken from four male rats. Before the *in vitro* fertilisation, sperm motility was analysed by using a computer-assisted sperm analyser (IVOS Sperm Analyzer, Hamilton-Thorne Research Co. Ltd., USA). Sperm were incubated in mHTF for 120 minutes at 37 °C under 5% CO2 in air and analysed using the IVOS system. We analysed the following sperm motility parameters: percentage of motile sperm (motile sperm that moved more than 5 µm/s), percentage of motile sperm with progressive motility (motile sperm with progressive motility were denoted by a path velocity >50 µm/s and a straightness ratio >50%) and a marker of hyperactivation [lateral amplitude of head (ALH): this is the average value of the maximum swing width of the sperm head]. In addition, path velocity (VAP), progressive velocity (VSL), beat frequency (BCF) and straightness (STR) were measured. Approximately 500 sperm were analysed in each experiment. In the case of cryopreserved sperm, sperm suspensions with greater than 30% total motility and 10% progressive motility measured via computer-assisted sperm analysis (Integrated Visual Optical System, Hamilton Thorn Inc., Beverly, MA, United States) prior to insemination were used for *in vitro* fertilisation (Supplementary Movie 1).

*In vitro* fertilisation was performed following procedures described previously^19,20^. Immature females were induced to ovulate via injections of 30 IU equine chorionic gonadotropin (ASKA Pharmaceutical Co. Ltd, Japan) and 30 IU human chorionic gonadotropin (hCG, ASKA Pharmaceutical Co. Ltd, Japan), which were administered 54–56 hours apart. Between 15–16 hours after the hCG injection, females were sacrificed via cervical dislocation, their oviducts were promptly collected, and all intact cumulus oocyte complexes were released from the collected oviducts into the preincubated sperm suspension (sperm concentration: 500–1200 cells/μL) for insemination. As a control, superovulated oocytes obtained using the same method were introduced to a fresh sperm suspension and were incubated for 2 hours (sperm concentration: 500 cells/μL).

Twenty hours after insemination, the oocytes were observed under an inverted microscope, and fertilisation rates were calculated as the total number of fertilised oocytes (oocytes with either two pronuclei and a sperm tail or with a sperm tail in the cytoplasm were judged as fertilised oocytes) were divided by the total number of inseminated oocytes and multiplied by 100.

In each experiment, 20 fertilised oocytes were transferred into the oviducts of ICRL:CD (SD) females (ten embryos/oviduct) on the day on which a vaginal plug was identified (day one of pseudopregnancy); embryos were transferred through the wall of the oviduct^21^. After 22–23 days, the number of pups was recorded. The remainder of the fertilised oocytes were cultured for an additional eight hours, and 100 of the fertilised oocytes that had developed to the 2-cell stage were cultured to the blastocyst stage in mR1ECM^22^. GFP signals in developed blastocysts were observed under a fluorescence microscope (BioRevo BZ-9000, Keyence Co. Ltd., Japan).

### Statistical analysis

Statistical analysis was performed using Prism version 5.0 (GraphPad). The results are expressed as the mean ± standard deviation. Group results were compared using Student’s t-test after arcsine transformation of the percentages; P < 0.05 was considered statistically significant.

## Supplementary information

Supplementary movie 1.
