# Author Correction: Establishment of sperm cryopreservation and *in vitro* fertilisation protocols for rats

**DOI:** 10.1038/s41598-020-60648-5

**Published:** 2020-02-25

**Authors:** Naomi Nakagata, Nobuyuki Mikoda, Satohiro Nakao, Ena Nakatsukasa, Toru Takeo

**Affiliations:** 1 Center for Animal Resources and Development, Kumamoto University, 2-2-1 Honjo, Chuo-ku, Kumamoto 860-0811 Japan; 2 Department of Animal Model Development, Brain Research Institute, Niigata University, 1-757 Asahimachidori, Chuo-ku, Niigata 951-8585 Japan

Correction to: *Scientific Reports* 10.1038/s41598-019-57090-7, published online 09 January 2020

The Article contains a typographical error in the Methods section under subheading ‘Sperm freezing’.

“A male rat was euthanised via cervical dislocation, and then its two cauda epididymides were removed aseptically and transferred into a 300 μL drop of CPA in a 35 mm culture dish at room temperature (Fig. 4A).”

should read:

“A male rat was euthanised via cervical dislocation, and then its two cauda epididymides were removed aseptically and transferred into a 3000 μL drop of CPA in a 35 mm culture dish at room temperature (Fig. 4A).”

